# Gastric Volvulus and Wandering Spleen: A Rare Surgical Emergency

**DOI:** 10.1155/2013/561752

**Published:** 2013-02-07

**Authors:** Georgios Lianos, Konstantinos Vlachos, Nikolaos Papakonstantinou, Christos Katsios, Georgios Baltogiannis, Dimitrios Godevenos

**Affiliations:** Division of Surgery, University Hospital of Ioannina, St. Niarchou Avenue, 45110 Ioannina, Greece

## Abstract

Gastric volvulus is a rare but potentially life-threatening clinical entity due to possible gastric necrosis. A wandering spleen may also be associated with gastric volvulus. Patients presenting with the triad epigastralgia, vomiting followed by retching, and difficulty or inability to pass a nasogastric tube into the stomach are likely to have gastric volvulus. The operating surgeon should include this rare entity in the differential diagnosis when dealing with a patient with such a clinical profile. Herein, we present a case of gastric volvulus associated with a wandering spleen in a 28-year-old Caucasian woman and we provide a brief review of the literature on this issue.

## 1. Introduction

 Gastric volvulus is an extremely rare clinical entity first described by Berti in 1866 [1]. An abnormal rotation (180°) of one part of the stomach around another, potentially leading to obstruction of the gastric cavity is defined gastric volvulus. Volvulus may be organoaxial or mesenteroaxial, occuring around an axis made by two fixed points. When untreated, complete volvulus results in strangulation, which may lead to ischaemia, necrosis, and finally to gastric perforation. Interestingly, mortality rates can achieve levels of 30–50% [2, 3]. Therefore, gastric volvulus is a true and life-threatening surgical emergency if not treated in time. Wandering spleen is also a rare entity characterized by the underdevelopment or complete absence of one or all of the ligaments that hold the spleen in its normal position. Moreover, gastric volvulus and wandering spleen share a common cause, the absence of intraperitoneal visceral ligaments [4, 5]. We hereby present the rare case of a 28-year-old Caucasian woman with gastric volvulus associated with a wandering spleen. We share our experience in successful treatment of this unique case.

## 2. Case Report

**Figure 1:**
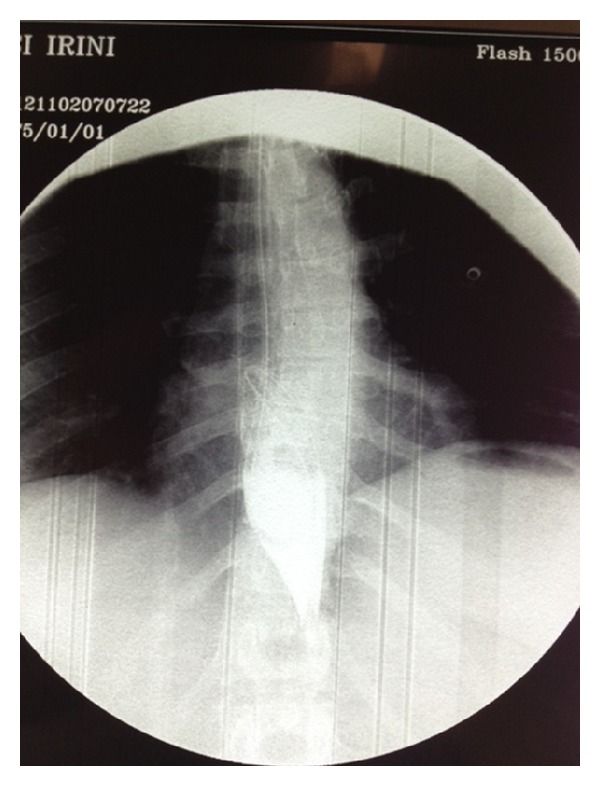
“Bird beak” sign and helical trend of the nasogastric tube after gastrografin swallow.

**Figure 2:**
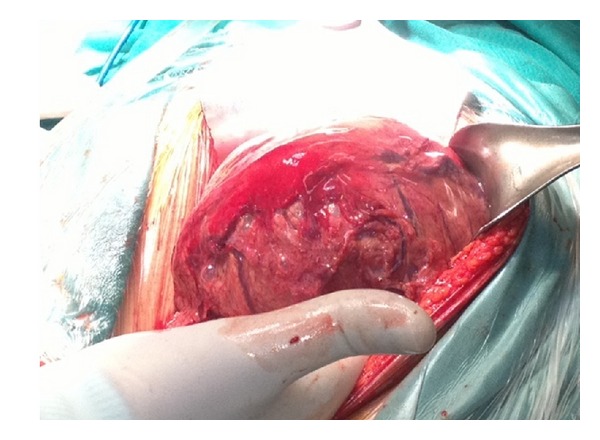
Twisted stomach (gastric volvulus).

**Figure 3:**
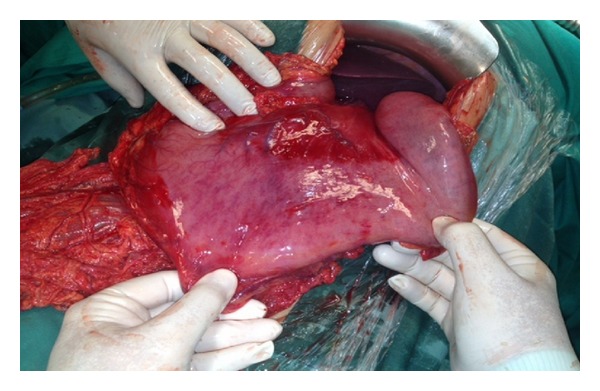
The untwisted distended stomach with ischemic mucosal lesions.

**Figure 4:**
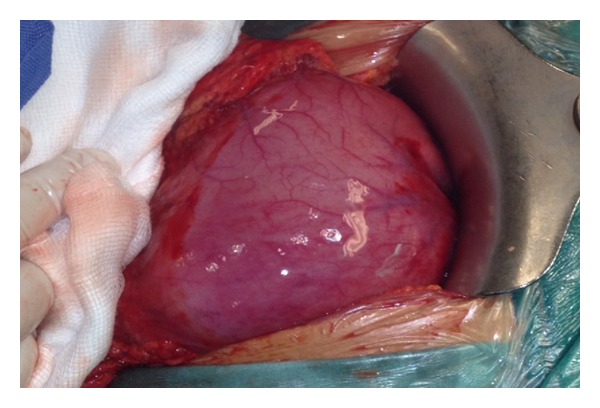
The untwisted distended stomach with ischemic mucosal lesions.

**Figure 5:**
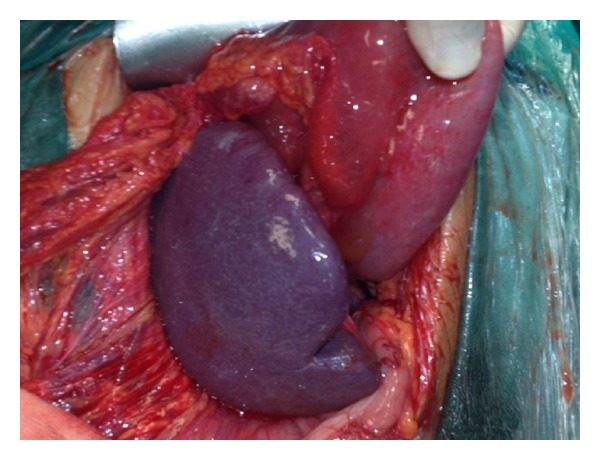
The wandering spleen.

**Figure 6:**
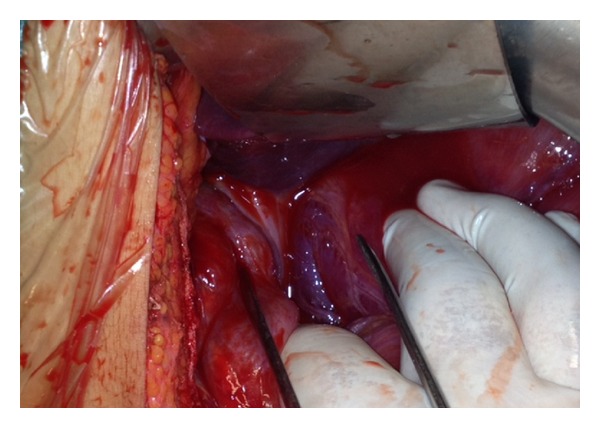
The approximation of the gastroesophageal junction and pylorus, leading to gastric volvulus.

**Figure 7:**
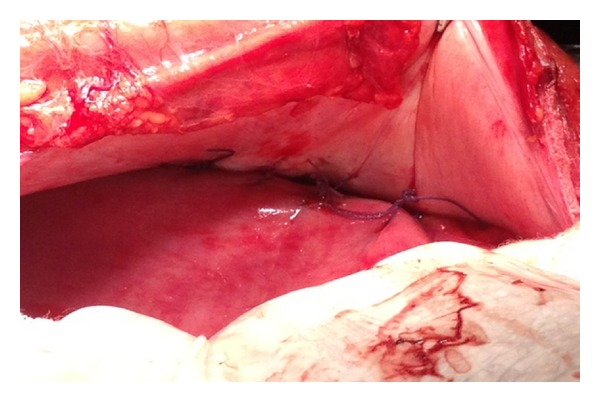
Anterior gastropexy.

A 28-year-old Caucasian female was admitted to the emergency department of our hospital complaining of severe abdominal pain, nausea, and multiple episodes of bilious vomiting followed by repeating nonproductive retching. Her medical history was unremarkable. Her vital signs showed only mild tachycardia. Upon physical examination the abdominal sounds were present and her abdomen was diffusely tender especially in the upper quadrant. No peptic ulcers or diaphragmatic hernias were included in her family history. Rectal examination showed an empty rectum. The laboratory tests were between normal ranges. There was a significant difficulty in passing a nasogastric tube which finally suctioned out a large amount of gastric fluid. The admission chest X-ray showed an enlarged stomach and an elevated left hemidiaphragm; so a gastrografin swallow was arranged. The latter showed a well-defined “bird beak” sign and a helical trend of the nasogastric tube (Figure 1). Gastroscopy was performed and revealed a large amount of gastric fluid in the stomach and mucosal ischemic lesions. Interestingly, the endoscope failed to intubate the pylorus. An exploratory laparotomy was performed under general anesthesia the day after by midline incision. Intraoperative findings revealed a twisted distended stomach with ischemic lesions. A mesenteroaxial gastric volvulus was identified (Figures 2, 3, and 4 ). Interestingly, there was a lack of ligaments; so a wandering spleen was observed (Figure 5). The volvulus was untwisted and an approximation of the gastroesophageal junction and pylorus, predisposing to volvulus, was revealed (Figure 6). The stomach was reduced at its anatomic position, as well as the spleen, and an anterior gastropexy was carried out by fixing the greater curvature of the stomach to the anterior abdominal wall (Figure 7). The postoperative period was uneventful and the patient was discharged 10 days later. Two months after the operation, the patient remained asymptomatic.

## 3. Discussion

Gastric volvulus is a rare entity with difficult diagnosis. The incidence is unknown [6]. An abnormal rotation of one part of the stomach around another of more than 180 degrees is defined gastric volvulus [7]. This infrequent entity is in almost all the cases associated with congenital diaphragmatic hernia and eventration of the diaphragm. Interestingly, there is also a rare association between gastric volvulus and wandering spleen. These entities share a common cause, the absence or laxity of intraperitoneal visceral ligaments. Wandering spleen is a mobile spleen attached only by its vascular pedicle. This spleen can migrate to any part of the abdomen [8]. Gastric volvulus can be acute, chronic, or acute on chronic. Acute gastric volvulus is more rare than chronic. There are described 3 types of gastric volvulus according to the axis of rotation: organoaxial, mesenteroaxial, and combination. Organoaxial volvulus is the most common and occurs in approximately 59% of cases. Because the duodenum and gastroesophageal (GE) junction are relatively fixed, the stomach rotates around the longitudinal axis extending from the gastroesophageal junction to pylorus. The mesenteroaxial volvulus is presented in 29% of cases. The rotation occurs around the transgastric axis (a line connecting the middle of the lesser curvature with the middle of the greater curvature). It is reported that most cases of chronic gastric volvulus are related to mesenteroaxial rotation. Additionally is described in the literature that a normal stomach cannot rotate more than 180° unless the gastrosplenic or gastrocolic ligaments are divided [9, 10]. The aetiology of gastric volvulus is thought to be secondary to laxity or lack of the gastric (gastrohepatic, gastrosplenic, gastroduodenal, and gastrophrenic) ligaments, allowing approximation of cardiac and pyloric ends when the stomach is full, leading to volvulus, as in our case (Figure 7) [11]. The clinical picture of gastric volvulus may occur as an acute abdominal emergency or as recurrent volvulus. In the literature, the famous Borchardt triad that is present typically in cases of acute gastric volvulus is described. This triad includes severe epigastric pain with distention, vomiting followed by violent, nonproductive retching, and finally difficulty or inability to pass a nasogastric tube into the stomach. Moreover, a missed diagnosis of gastric volvulus may lead to strangulation, perforation, hemorrhage, ischemia, and gastric necrosis [12]. The mortality rate of gastric volvulus is reported to be up to 42–56%, secondary to gastric ischaemia, perforation, and necrosis [13]. The diagnosis of this rare clinical entity is very challenging. The gold standard method in detecting gastric volvulus is a barium swallow, which has a very high sensitivity and specificity. Additionally, highly suggestive of gastric volvulus is the difficulty during endoscopy to intubate the stomach or the pylorus. As for the treatment, gastric volvulus presenting with acyte symptoms requires immediate surgical intervention. The most approved surgical treatment consists in anterior gastropexy with open or laparoscopic technique. During this method, the greater curvature of the stomach is fixed to the anterior abdominal wall. Subtotal or total gastrectomy is proposed when the stomach appears gangrenous [14, 15].

## 4. Conclusion

Though rare, gastric volvulus must be always considered in the differential diagnosis when a patient with the Borchardt triad is admitted to the hospital. A missed or delayed diagnosis may result in unfavorable outcomes. It seems that the most important factor in diagnosing gastric volvulus is the awareness of its possibility. The diagnosis is suspected mainly by symptoms and exclusion of other pathologies. Surgical intervention is the optimal treatment. Additionally, gastric volvulus is rarely associated with wandering spleen that may lead to splenic torsion. These entities are potentially life-threatening, if not treated in time.
